# Feasibility of a quality-improvement program based on routinely collected health outcomes in Dutch primary care physical therapist practice: a mixed-methods study

**DOI:** 10.1186/s12913-024-10958-5

**Published:** 2024-04-24

**Authors:** LSF Smeekens, AC Verburg, MJM Maas, R van Heerde, A van Kerkhof, PJ van der Wees

**Affiliations:** 1 Scientific Institute for Quality of Healthcare, Radboud university medical center, Kapittelweg 54, 6525 EP Nijmegen, The Netherlands; 2 HAN University of Applied Sciences, Nijmegen, The Netherlands; 3 Leidsche Rijn Julius Gezondheidscentra, Utrecht, The Netherlands

**Keywords:** Nonspecific low back pain, Health outcomes, Physical therapy, Quality improvement

## Abstract

**Background:**

This study evaluates the feasibility of a nine-month advanced quality-improvement program aimed at enhancing the quality of care provided by primary care physical therapists in the Netherlands. The evaluation is based on routinely collected health outcomes of patients with nonspecific low back pain, assessing three feasibility domains: (1) appropriateness, feasibility, and acceptability for quality-improvement purposes; (2) impact on clinical performance; and (3) impact on learning and behavioral change.

**Methods:**

A mixed-methods quality-improvement study using a concurrent triangulation design was conducted in primary care physical therapist practice. Feedback reports on the processes and outcomes of care, peer assessment, and self-assessment were used in a Plan-Do-Study-Act cycle based on self-selected goals. The program’s appropriateness, feasibility, and acceptability, as well as the impact on clinical performance, were evaluated using the Intervention Appropriate Measure, Feasibility Intervention Measure, Acceptability Intervention Measure (for these three measure, possible scores range from 4 to 20), and with a self-assessment of clinical performance (scored 0–10), respectively. The impact on learning and behavioral change was evaluated qualitatively with a directed content analysis.

**Results:**

Ten physical therapists from two practices participated in this study. They rated the program with a mean of 16.5 (SD 1.9) for appropriateness, 17.1 (SD 2.2) for feasibility, and 16.4 (SD 1.5) for acceptability. Participants gave their development in clinical performance a mean score of 6.7 (SD 1.8). Participants became aware of the potential value of using outcome data and gained insight into their own routines and motivations. They changed their data collection routines, implemented data in their routine practice, and explored the impact on their clinical behavior.

**Conclusions:**

This explorative study demonstrated that a quality-improvement program, using health outcomes from a national registry, is judged to be feasible.

**Impact statement:**

This study provides preliminary evidence on how physical therapists may use health outcomes to improve their quality, which can be further used in initiatives to improve outcome-based care in primary physical therapy.

**Supplementary Information:**

The online version contains supplementary material available at 10.1186/s12913-024-10958-5.

## Background

High-quality health care is defined as care that is safe, timely, equitable, effective, efficient, and patient centered [1]. Against a background of rapidly increasing healthcare costs, service restrictions, and differences in quality, there is an increasing need for initiatives to improve quality of care [2]. This has led the Royal Dutch Society for Physical Therapy (KNGF) to initiate the ‘Quality in Motion’ program, which aims to improve the effectiveness and patient centeredness of care in physical therapist practice by providing therapists with feedback on health outcomes [3]. Outcome measures include patient-reported outcomes (PROs), which are used to assess aspects of a patient’s health status coming directly from the patient. Patient-reported outcome measures (PROMs) are questionnaires or single-item scales used to assess PROs [4], and can be used to support quality improvement [3]; however, there is a clear lack of understanding about how physical therapists can best utilize feedback about PROs to improve quality of care [5–7].

Nonspecific low back pain (NSLBP) is one of the most common health conditions in primary physical therapist practice [8, 9]. Based on health outcomes from a clinical registry and consensus among stakeholders (i.e., physical therapists, researchers, patients, and health insurers), Verburg et al. [4] developed a core set of PRO-based quality indicators for patients with NSLBP in primary physical therapist practice. The set was found to be useful for quality-improvement initiatives, and stakeholders reported that it added value for routine practice [3, 4]. These outcomes can be aggregated across patients in clinical registries, providing data for managing clinical quality, benchmarking and public reporting across organizations, and in clinical research; however, their aggregated use for quality improvement was found to be suboptimal [10–12]. An earlier study found that electronic health record (EHR) compatibility and therapist knowledge of the PROMs are the two key barriers to wider PROM use [13], with similar issues reported in other professions [14, 15].

Feedback interventions, particularly when provided by a colleague both verbally and in writing [16], have shown promise in improving physical therapist practice [17, 18]. Correspondingly, feedback reporting on processes and outcomes of care has been identified as an effective intervention that can support the exchange of best practices and mutual learning [16, 18, 19]. Additionally, involving peers as feedback providers in peer assessment creates meaningful learning experiences and is associated with behavioral change and measurable performance improvement in healthcare professionals [20–22]. Maas et al. [23] showed that peer assessment using video recordings of client communication and clinical records is an effective feedback intervention method in enhancing commitment to change and improving the clinical performance of physical therapists. Furthermore, feedback interventions seem to be more effective in changing clinical behavior when including clear targets and an action plan [16]. Accordingly, the Plan-Do-Study-Act facilitates systematic testing of changes in real-world settings, allowing for rapid learning and adaptation. This approach has been effectively utilized in various healthcare studies to enhance clinical outcomes and process efficiencies [24]; however, most physical therapists are not familiar with such quality-improvement interventions based on health outcomes [25].

The aim of this study is therefore to evaluate the feasibility of an advanced quality-improvement program for physical therapists in primary care. The evaluation involves feedback, peer assessment, and self-assessment in a rapid improvement Plan-Do-Study-Act cycle, using the routinely collected health outcome data of patients with NSLBP.

## Methods

### Study design and setting

The program feasibility was evaluated through an explorative quality-improvement study using a mixed-methods approach in a concurrent triangulation design [26]. The following program feasibility domains were addressed [27]: (1) appropriateness, feasibility, and acceptability for quality-improvement; (2) impact on clinical performance; and (3) impact on learning and behavioral change. We used the Standards for QUality Improvement Reporting Excellence (SQUIRE) Guidelines [28]. The evaluation was conducted between January and October 2022. We tested our program in a convenience sample of Dutch primary care physical therapists organized in a regional network of communities of practice (the Cooperation of Physical Therapists Nijmegen; CFN).

### Participants

All physical therapy practices within the CFN network (*n* = 30) were approached to recruit therapists for the study. Invitations were extended via a digital newsletter, which included the goals of the study and contact details of the first author (LS). Physical therapists willing to participate received detailed study information by email and were screened for eligibility using the inclusion criteria below. Participation was voluntary. All participants provided written informed consent.

### Inclusion criteria

Licensed Dutch physical therapists were eligible to participate in this study if they provided primary care to patients with NSLBP aged 18 years or older [3, 4]. They also had to evaluate selected outcomes as part of a standard clinical routine in patients with NSLBP using the following PROMs (associated domain): Numeric Pain Rating Scale (NPRS) (pain intensity), Patient Specific Functioning Scale (PSFS) (physical activity), Quebec Pain Disability Scale (QBPDS) (physical functioning), Global Perceived Effect (GPE-DV) (perceived treatment effect), and STarT Back Screening Tool (SBST) (profile grouping based on risk of poor outcome) [3, 4]. Physical therapists collected outcomes using these PROMs, which were directly recorded into their EHRs. These data were transferred to the national data registry of the Royal Dutch Society for Physical Therapy (KNGF). Additionally, to facilitate meaningful participation in the quality-improvement program, particularly during peer assessment sessions and outcome discussions, it was essential for participants to have contributed sufficient data to the national clinical registry from January 2021 to November 2021 (a minimum requirement of five patients with a closed treatment episode). An episode was considered closed when the physical therapist closed the episode in the EHR, or if six weeks had passed after the last visit. Informed consent for delivering data to the national clinical registry was obtained from every patient. This approach ensured that participants could engage with actual data reflective of their clinical practices rather than hypothetical scenarios, fostering deeper learning and reflection on professional conduct and patient care. The requirement for therapists to have already been actively collecting and submitting data as part of their clinical routine underlines the study’s aim to engage therapists who were not only familiar with the use of PROMs, but who also had sufficient data to enable a meaningful analysis and discussion within the context of the quality-improvement program.

### The quality-improvement program content

The nine-month program consisted of a rapid improvement cycle comprising multiple consecutive steps and quality-improvement interventions. In step 1, participants were offered the opportunity to complete an e-learning module on using data in clinical practice [29]. In step 2, personal data exports were extracted from the national clinical registry. Participants received feedback reports on the processes and outcomes of their care in step 3 [30–32], then attended peer assessment meetings in step 4 [18, 23, 33], In step 4, the therapists drafted a rapid improvement Plan-Do-Study-Act cycle and individual quality-improvement goals [6, 34, 35], and in step 5, they performed a self-assessment of their clinical performance [36]. See Additional File 1 for further details of the program. The process and outcome indicators of the PROMs for patients with NSLBP were used in the program (see Additional File 2) [3, 4].

### Evaluation of program feasibility and outcome measures

The program’s perceived appropriateness, acceptability, and feasibility for quality-improvement purposes were evaluated using the Dutch versions of the Intervention Appropriate Measure (IAM), the Feasibility Intervention Measure (FIM), and the Acceptability Intervention Measure (AIM), respectively [37], which have been demonstrated to be valid and reliable tools [37]. Each measure consists of four items scored on a five-point Likert scale, with higher scores indicating better appropriateness, acceptability, and feasibility, respectively (scoring range: 4–20 for each tool). The impact on clinical performance was evaluated using self-assessment checklists [36] (steps 5 and 7 of the quality-improvement program), while the impact on learning and behavioral change was qualitatively determined during the peer assessment (steps 4 and 6). We used a parallel approach in collecting the quantitative and qualitative data, giving equal weight to both methods.

### Data collection

Participants were invited by email to attend the peer assessment meetings. A script (see Additional Files 5 and 6) for each meeting was designed by the research team, addressing different quality-improvement interventions. A participatory evaluation strategy was used, allowing an assessment of the impact of the program on learning and behavioral change during the actual implementation [38]. The peer assessment meetings lasted 100–120 min and were conducted face-to-face by an external coach (RvH) using open-ended questions, which facilitated group discussion and knowledge development. A safe environment was encouraged within each peer group [20, 22]. The peer assessment meetings were audio-taped, video-recorded, and subsequently transcribed verbatim. Written informed consent was obtained from all participants. The identities of the participants were considered confidential; therefore, the transcripts of the meetings were processed anonymously. Participants were asked to complete a self-assessment checklist halfway through the program, at the end, and six months after via email. Likewise, participants completed the IAM, FIM, and AIM at the end of the study, following the second peer assessment meeting.

### Data analysis

#### Quantitative analysis

**Fig. 1:**
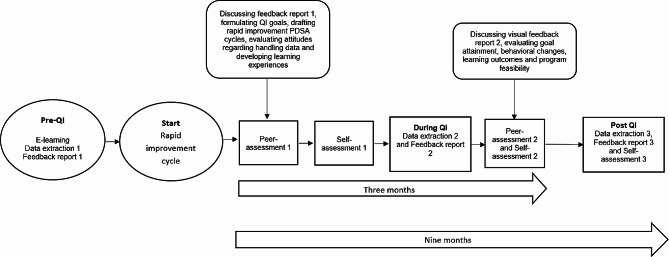
The structure of the quality-improvement (QI) program QI = quality improvement; PDSA = Plan-Do-Study-Act

The mean scores and standard deviations (SDs) of the IAM, FIM, and IAM were calculated. For the quality-improvement program to be considered appropriate, feasible, and acceptable [37], a minimum mean score of 15 out of 20, averaged over all participants, was required for each measure. The mean scores and SDs were calculated separately for the self-assessment checklists at three timepoints. For the quality-improvement program to be considered to impact the development of clinical performance, a minimum mean score of 5 out of 10 was required [36], averaged over all competed self-assessment checklists. Our comparative analysis focused on the mean scores and differences in process and outcome indicators between two periods: the pre-improvement period (the 12 months before the start of the study) and the quality-improvement period (the nine months after the study began). The latter period integrates data from both the initial and subsequent phases of the quality-improvement program, reflecting insights consolidated from the two feedback reports received by the participants during the program (Fig. 1). Our analysis focused exclusively on complete case episodes with both baseline and endpoint measurements to ensure the integrity and applicability of the data for participation in the quality-improvement program. All quantitative data were analyzed using SPSS Statistics, version 26 (IBM, Armonk, New York, USA).

#### Qualitative analysis

Transcripts of the peer assessment meetings were read in detail, and a directed content analysis was used to study them [39, 40]. A codebook was developed in advance, informed by the research questions. Text fragments were labeled according to these *a priori* codes, which were further refined during the coding process. Meaningful text fragments that could not be labeled were coded inductively. The transcript analysis was supported by ATLAS.ti version 8.4 [41]. Two researchers (LS and AvK) independently coded the transcripts, discussed the codes to reach consensus, and created the codebook, allocating codes into categories based on their similarities [42]. A researcher (MM) with ample experience in peer assessment and qualitative research guided this process. Kirkpatrick’s model, which was designed to evaluate the impact of an educational program, was used to allocate the identified categories to four domains: reaction, learning, behavior, and results (see Additional File 7) [43]. Preliminary findings after both peer assessment meetings and the final codes, categories, and the allocation of categories to the domains were discussed by the research team (LS, MM, RvH, AV, and PvdW) in several meetings. A member checking procedure was conducted by sending a summary with preliminary results to all participants after the first meeting to increase the credibility of the results. To optimize the transferability of the results, we aimed to saturate the information by recruiting at least three peer groups.

## Results

In total, 10 physical therapists from two different practices participated in the program. Two mixed-practice peer groups were formed, each consisting of five participants. The participants’ characteristics are outlined in Table 1.

**Table 1:** Characteristics of participating physical therapists (*N* = 10)

Characteristics	
Age in years, mean (SD)	34.1 (9.5)
Men: women (%)	9:1 (90:10)
Experience in years, mean (SD)	10.4 (9.6)
Educational status	
*Bachelor, n (%)*	7 (70)
*Masters degree, n (%)*	3 (30)
Successfully completed the e-learning, n (%)	9 (90)

N = Number of participants; SD = standard deviation

### Quantitative results

Table 2 provides an overview of the appropriateness, feasibility, and acceptability of the program, as well as the perceived development in clinical performance. All predefined criteria regarding the minimum score on the IAM, FIM, AIM, and the self-assessment checklists were met.

**Table 2:** Mean scores of all participants on the IAM, FIM, AIM, and the self-assessment checklists

Mean (SD) Fulfilling the criteria (Yes/No)		
IAM*	16.5 (1.9)	Yes^†^
FIM*	17.1 (2.2)	Yes^†^
AIM*	16.4 (1.5)	Yes^†^
Self-assessment checklist during QI (six weeks after start) ^‡^	6.1 (1.8)	N.A.
Self-assessment checklist at the end of QI (three months after start) ^‡^	7.3 (1.7)	N.A.
Self-assessment checklist post-QI (nine months after start) ^‡^	7.3 (1.6)	N.A.
Total average on all self-assessment checklists^‡^	6.9 (1.7)	Yes^§^

IAM = Intervention Appropriate Measure; FIM = Feasibility Intervention Measure; AIM = Acceptability Intervention Measure; SD = standard deviation; QI = quality improvement. ^*^ Scores range from 4–20; a higher score indicates a greater appropriateness, feasibility, and/or acceptability. ^†^For the QI program to be judged as appropriate, feasible, and acceptable, a minimum mean score of 15 out of 20, averaged over all participants, on each questionnaire (AIM, FIM, or IAM, respectively) was required. ^‡^Scores range from 0–10; a higher score indicates more development in clinical performance. ^§^For the QI program to be judged as impacting the development of clinical performance, a minimum mean score of 5 out of 10, averaged over all competed self-assessment checklists, was required

The mean process and outcome indicator scores for the three data periods are compared in Table 3. All process indicators improved substantially during and after the quality-improvement cycle, with mean improvements ranging from 9 to 26%.

**Table 3:** Mean scores of all participants on the process and outcome indicators and the mean differences

Type of indicator	PROM	Pre QI, mean (SD) (*N* = 186) ^††^	During and post QI, mean (SD) (*N* = 175) ^††^	Mean difference (SD)
Process (0–100) (%)	NPRS*	54% (24.1)	75% (19.1)	21% (28.9)
Process (0–100) (%)	PSFS*	59% (29.1)	75% (19.6)	16% (31.7)
Process (0–100) (%)	QBPDS*	43% (22.9)	69% (20.1)	26% (28.0)
Process (0–100) (%)	GPE-DV^†^	48% (26.3)	70% (21.6)	22% (30.4)
Process (0–100) (%)	SBT^‡^	83% (12.1)	93% (7.3)	9% (10.8)
Mean change (0–10)	NPRS^§^	5.5 (2.4)	5.6 (0.7)	0.03 (2.3)
Mean change (0–10)	PSFS^§^	5.5 (2.1)	6.1 (1.3)	0.6 (1.6)
Mean change (0–100)	QPBDS^§^	27.2 (15.3)	28.5 (9.4)	1.3 (14.4)
Mean score (1–7^#^)	GPE-DV ^||^	1.9 (0.7) ^**^	1.8 (0.5) ^**^	0.1 (0.4) ^**^

QI = quality improvement; PROM = patient-reported outcome measure; SD = standard deviation; NPRS = Numeric Pain Rating Scale; PSFS = Patient Specific Functional Scale; SBST = STarT Back Screening Tool; QBPDS = Quebec Back Pain Disability Scale; GPE-DV = Global Perceived Effect—Dutch Version. *The process indicator reflects a repeated measurement; pretreatment and posttreatment. ^†^The process indicator reflects a single measurement; posttreatment. ^‡^The process indicator reflects a single measurement; pretreatment. ^§^The outcome indicator reflects the change score between a pretreatment and posttreatment measurement. ^||^The outcome indicator reflects a single posttreatment measurement. ^#^1 (absolutely satisfied) to 7 (absolutely unsatisfied). ^**^The average reflects the scores of seven participants, as GPE-DV outcome scores were not accessible in the clinical registry for three participants. ^††^Reflects the number of closed treatment episodes on which process and outcome indicators are based

### Qualitative results

We conducted four peer assessment meetings, two for each peer group. After analyzing the qualitative data, the codes were classified into eight major categories. These categories were allocated to the four domains of Kirkpatrick’s model of evaluation (see Table 4). Quotes are numbered and labeled by peer group (see Table 5).

**Table 4:** Categorization of domains, major categories, and topics

Domains according to Kirkpatrick [43]	Major categories	Topics
Reaction	1) Program appreciation	Novelty, Meaningfulness, Pleasurability, Acceptability, Burden
	2) Suggestions for program improvement	Suggestions for the improvement of the program content
Learning	3) Awareness and insight	Awareness of existence and possibilities for examining data
		Awareness of opportunities to compare, clarify, and implement data
		Awareness of the importance and value of data collection
		Interpreting, clarifying, and understanding data
		Insight and recognition of data collection and implementation routines
		Acknowledgment of the importance of appropriate data collection routines
		Acknowledgment of potentially valuable areas for implementing data
		Acknowledgment of potential value of data for improving quality of care
	4) Motivational change	Data collection motivations
		Priority of data collection
Behavior	5) Intentions for behavioral change	Improving process indicators
		Changing data collection routines
		Implementing data to support clinical behavior
	6) Demonstrated behavioral change	Revision of the extent of collected data
		Revision of the manner of data collection
		Implementing data to support clinical behavior
		Communicating data collection to patients
	7) Barriers and facilitators	Barriers to accomplish intended behavioral changes
		Facilitators for accomplishing intended behavioral changes
Results	8) Goal attainment	Perceived extent of reaching goals
		Extent of improvements in process indicators
		Extent of changes in data collection routines

**Table 5:** Quotes of participants

Reaction	
Q1-G2	*“Yes, I thought [participating in the program] was very meaningful. For me, it was quite an eye-opener. I think I did a lot with it. I hope to see the results in a moment. But very purposeful, yes.”*
Q2-G1	*“Maybe [the program should] indeed incorporate more emphasis on the goal of collecting data and the clinical relevance you can gain from it, because this will stimulate awareness among physical therapists. Like, the ‘why’ - why do you [collect data] the way you do?”*
Q3-G1	*“I personally think it would be more interesting to repeat [the second peer assessment] and explore the outcomes that appear then. This [study] period is maybe a little too short to draw firm conclusions from the outcomes.”*
**Learning**	
Q4-G1	*“I think it is certainly valuable to experience as a young physical therapist that data exist and that there are comparison materials available, that you can form an opinion about this and search for explanations. For that matter, it is really good to learn to think in this way.”*
Q5-G1	*“But I think that we might be very critical; can you draw conclusions from the data? Because if we are already remarking how large discrepancies exist between the methods of data collection and what desirable outcomes we are actually filling in for patients, then we can ask ourselves the question: are these data useful at all?”*
Q6-G1	*“Yes, [measuring the actual outcome of the patient] is the most ideal situation, as it were. Eventually however, especially in repeated measurements, it is often the case that you have an end evaluation and you are busy and you ask the patient ‘can I send the questionnaire online’? And if this questionnaire is not completed, you have to close the treatment episode [by completing it yourself]. That’s kind of what it comes down to in daily practice, and you score higher then.”*
Q7-G1	*“I thought of this as a painful conclusion. We ask our patients to complete a questionnaire, which requires around 20 min of their time, while we do not take the measure completely seriously and actually just…don’t use it as an input to guide future treatment…”*
Q8-G2	*“I said let’s perform an evaluation measurement and based on that we moved in another direction, and are going to evaluate again in six weeks. So, if we are talking about quality of care for the patient, I think I’m more consciously working to improve that.”*
Q9-G2	*“Yes, and a less obligatory mindset and more being allowed to and wanting to improve care. Because I think most people in this room want to improve and develop their skills and treatments or whatever. I think that…this shift is being accomplished, from ‘we have to’ complete the questionnaires to ‘I want to’ complete them…”*
**Behavior**	
Q10-G2	*“I formulated three goals. 1. Completing the start and end measurements more often, purely quantitative… 2. Using [end measurements] more often as a tool to decide whether the treatment episode [should] be closed or continued, together with the patient. And 3. leaving the completion of questionnaires to patients more frequently.”*
Q11-G2	*“In the repeated measurement I tell [the patient] ‘you scored a certain number of points on this measure last time for this activity, so where are you now?’ And the patient will score the activity again and subsequently we can reflect on that together.”*
Q12-G1	*“I still barely use [the collected data] to guide my treatments. I only use them when closing a treatment episode actually… I’m not using it to gain something extra out of it; I obtained this information with my own history taking and physical examination.”*
Q13-G2	*“It is time consuming…Let’s say you have a completed QBPDS and the EHR system could…select items that stand out, then you might implement or apply it more. But currently I have to interpret the whole questionnaire myself and what comes out of it. So [automated data analysis] would help me.”*
Q14-G2	*“That QBPDS is just really a Quebec Pain in the Ass, because it is a really time-consuming and long questionnaire…”*
Q15-G1	*“If we are really being honest, time is the most precious thing there is. Look, if we ask our patients to complete a questionnaire and it will take them 20 min and we do nothing with it, we can ask ourselves what are we really doing? Actually, that is a little odd. Sometimes this makes you think that we only do it because we have to. So that relevance of why you should [collect outcome data] needs to be much clearer in advance. I think that is very important. I think a lot of physical therapists actually don’t know that.”*
**Results**	
Q16-G2	*“I don’t know exactly how much [I improved], but I think it is not realistic to improve [on process indicators] as much as I intended, as some people suddenly stop treatment and it is very difficult to contact them sometimes.”*
Q17-G1	*“Yes, we complete more questionnaires, but for me it is important that it is not just done for show. Yes, it is completed more, but [we must also do] it with more intrinsic motivation and not solely because of external pressure to participate in good practice or from practice owners; you actually do it to improve quality of care.”*

Q = quote number; G = peer group number; QBPDS = Quebec Back Pain Disability Scale; EHR = electronic health system; NPRS = Numeric Pain Rating Scale; PSFS = Patient Specific Functioning Scale

### Domsain: reaction

#### Program appreciation; suggestions for program improvement

Participating in a quality-improvement program based on routinely collected health outcomes was novel for most participants. In general, the therapists considered the program’s content meaningful, pleasant, acceptable, and accessible (Q1-G2), and proposed several advancements to increase future program experiences and satisfaction (Q2-G1)(Q3-G1).

### Domain: learning

#### Awareness and insight

Most participants became more aware of the existing data and the possibilities for analyzing and comparing them. They developed an understanding of the clinical relevance of the data presented, and identified possible explanatory factors by interpreting and clarifying the data (Q4-G1). Participants also gained insight into how to appropriately design data collection, the importance of proper data collection methods (Q5-G1), and potential areas for implementing data in routine practice.

Participants became more aware of data collection throughout the quality-improvement cycle, but acknowledged the lack of a standardized, valid, and reliable data collection method (Q6-G1). Before the quality-improvement program, most participants did not routinely use data to guide and improve their practice, despite dedicating considerable effort to its collection (Q7-G1).

The evolving knowledge gained from the quality-improvement cycle led participants to realize that routinely implementing data can enhance their clinical practice, and more importantly can significantly benefit patients (Q8-G2). Some participants openly argued that using data will not improve the quality of their physical therapy. They challenged the perceived value of the data in comparison with their own expertise and discussed the required time investment in relation to the perceived returns.

#### Motivational change

Collecting and using data with the objective of improving quality of care for the patient was not a common mindset among participants. Instead, data collection was performed to meet obligatory external requirements and was not considered a priority. However, as the quality-improvement cycle continued, most participants reported a shift to more intrinsically motivated efforts for collecting data (Q9-G2).

### Domain: Behavior

#### Intentions for behavioral change

Participants were encouraged to reflect on their own clinical behavior and reported feeling motivated to change their routine practice. All participants planned to improve their process indicators and data collection routines, particularly by allowing patients to complete their own questionnaires. Some participants proposed integrating the data into their practice and investigating its impact on their clinical behavior (Q10-G2).

#### Demonstrated behavioral change

All participants revised the extent and approach of their data collection. Most participants successfully applied some form of data use in routine practice, such as to evaluate treatment progress, to guide treatment and decision-making processes, as input for taking patient histories, for patient empowerment, for goal setting with the patient, and to complement or contradict their own assumptions (Q11-G2). Although they changed their data collection routines, two participants admitted they still rarely used data to support their clinical behavior (Q12-G1).

#### Barriers to and facilitators of behavioral change

Participants identified several barriers and facilitators that hindered or helped them to achieve their intended behavioral changes (Q13-G2)(Q14-G2)(Q15-G1) (see Table 6). These factors impacted the quantity of data collected, influenced the data collection protocols used, and shaped efforts to integrate data into routine practice.

**Table 6:** Barriers and facilitators for accomplishing the intended behavioral changes

Barriers
*Time investment, gathering endpoints, and patient needs*: the length and total volume of the MDS for patients with NSLBP, gathering endpoint measures in the absence of patients, not meeting patient expectations
*External pressure*: the need to meet external requirements regarding scores on process and outcome indicators, stimulating inappropriate ways of collecting data; noticeably formalized to just ‘fulfill’ these demands
*Financial aspects*: narrow additional insurance, limited time available within these budgets is preferably spent treating the patient at the expense of collecting data
*Population characteristics*: lack of digital skills and low (health) literacy of patients constrain legitimate data collection
*Irrelevant content, overlap between and scope of questionnaires*: inappropriateness of questions and applicability for every treatment profile, existence of overlay in questions and measures of the MDS regarding physical activity and functioning
*EHR system specifications*: lack of a well-functioning EHR system and specifications to guide online data collection, lack of external reminders for data collection
*Lack of knowledge and skills*: how to interpret (clinical relevance) and how to apply data to support clinical behavior
**Facilitators**
*Goal-setting; functional, specific, and concise instruments*: relevant aspects for the patient, short and easy to apply questionnaires, dynamic questionnaires; computer-assisted testing (CAT)
*External reminder for data collection (future assurance)*: external reminder to complete data collection in open treatment episodes, through a quality manager or the EHR system
*Awareness of the added value of data collection to improve quality of care*: more knowledge of the why and relevance of data collection may stimulate behavioral change, including communicating the value of data collection to patients
*Education on how to handle the collected data to support clinical behavior*: more attention paid to data collection routines (including EHR aspects) and handling data in routine practice during the undergraduate education of physical therapists
*Overview of results*: making the most relevant and eye-catching outcomes of completed questionnaires clearly visible (EHR system)
*Peer assessment atmosphere*: open and safe environment and incentives to reflection through open-ended questioning
*Rapid improvement nature of the program*: enhanced involvement and awareness regarding the transfer-of-learning

MDS = minimum data set; NSLBP = Nonspecific Low Back Pain; EHR = electronic health record

### Domain: results

#### Goal attainment

The majority of participants set goals related to processes and collection routines. Seven of the 10 participants accomplished their personal targets regarding improving process indicators (Q16-G2). All participants achieved their objectives around changing data collection routines. One participant openly debated the benefit of goal attainment on the added value and quality of care for the patient (Q17-G1).

## Discussion

This study explored the feasibility of a quality-improvement program designed to enhance the quality of primary care physical therapists. The program uses health outcomes from a national registry and incorporates feedback, peer assessment, and self-assessment in a Plan-Do-Study-Act cycle. We found that the participants considered the program an appropriate, feasible, and acceptable intervention for quality-improvement purposes, and found it beneficial for improving their clinical performance. All participants improved the completeness of the data they collected. They also gained insights into the potential value of using outcome data in clinical practice, as well as in examining their routines and motivation. Participants recognized the importance of handling data, revised their data collection methods, began to implement data use into their routine practice, and observed the impact on their clinical behavior. They acknowledged the added value of using data when formulating clear treatment targets, monitoring treatment processes, motivating patients, and, on an aggregated level, improving the quality of care. While most participants reacted positively to the program and acknowledged its added value, they faced significant challenges, such as the complexity of integrating systematic data collection into daily practice, external pressures to meet specific outcome benchmarks, and the need for more knowledge and skills in data interpretation and application. These factors sometimes hindered the full realization of the program’s benefits and highlighted areas for improvement that should be addressed to improve the program before wider implementation.

### Comparison to similar studies

This study builds upon previous research that highlighted the potential value of outcome data in quality-improvement initiatives [4, 24]. When evaluating the potential value of feedback, peer assessment, self-assessment, and Plan-Do-Study-Act cycles in physical therapist care, most previous studies did not use aggregated real-world data from clinical registries. Maas et al. [23] and Steenbruggen et al. [36] incorporated feedback, peer assessment, and self-assessment in comprehensive quality-improvement programs aimed at the professional development of physical therapists, using client records, video recordings of client communication, and the tracer methodology, respectively. Both programs were found to be feasible and led to improvements in clinical performance [23, 36]. The results of the present study support and extend previous findings of these quality-improvement strategies in physical therapist practice.

During the initial peer assessment meeting, the participants gained new knowledge and became more conscious of their own behavior. These findings are consistent with previous research indicating that peer assessment promotes learning, increases self-awareness [22, 44], and builds self-concept [45, 46]. Additionally, participants developed a critical perspective regarding their daily routines and expressed a desire to change their behavior. A similar enhanced commitment to change was reported by physical therapists who underwent cycles of peer assessment and self-assessment [23]. These findings are in line with theories of health behavior, which suggest that all behavioral change begins with recognizing one’s own behavior [47], and with the intention to change [48].

Another important finding was the observed shift in motivation for collecting data. Prior to the quality-improvement program, data were often collected in a non-validated manner, driven by external factors such as health insurers, and were not used to improve patient care. This is consistent with previous findings that the use of feedback in quality improvement is hindered by a perceived political motive for public reporting rather than improved patient care [7], by financial incentives from health insurers [49], and by a lack of experience and skills [7, 50]. Instead of collecting data to meet an external goal, most participants moving along the quality-improvement cycle reported a shift to a more intrinsic motivation. This could be attributed to participants giving new meaning to collecting and handling data in their daily practice, and establishing their own personal values. These findings are consistent with Ryan and Deci’s self-determination theory, which states that the basis for intrinsic motivation and behavior is formed by people finding a rationale within themselves [51]. Indeed, participants in the current study emphasized the importance of having clear self-directed motives for data collection as a key driver of behavioral change. Consistent with this, healthcare providers previously reported being more likely to take steps for quality improvement in response to the feedback of aggregated PROMs if they perceived these data to be credible and beneficial for improving patient care [19]. Throughout the quality-improvement cycle, learning and understanding of data management continued to be developed through experience and reflection, in line with Dewey’s experiential learning theory [52].

All participants made self-initiated behavioral changes during the program, which was believed to be supported by the application of knowledge gained by following the Plan-Do-Study-Act cycle [24]. Setting specific targets and making an action plan may increase the effectiveness of feedback and facilitate behavioral changes [18]. In the present study, feedback was provided by a colleague, more than once, both verbally and in writing to further increase its effectiveness. The participants were largely successful in changing their data-collection procedures; however, there is still room for improvement in the use of data in routine daily practice. Previous studies have shown that clinicians find PROMs useful for supporting the therapeutic process [19]; however, it took more time or effort to develop these application skills than was available within the timespan of the program. This assumption is supported by the feedback intervention theory, which assumes that the effectiveness of feedback is lower when the ‘task novelty’ and ‘task complexity’ are higher [53]. Indeed, participants mentioned a lack of knowledge and skills regarding data application as important barriers to its use. Feeling competent is very important for accomplishing behavioral change, according to the self-determination theory [51]. Correspondingly, previous research indicated that healthcare providers need more support and guidance on how to structurally implement data into their daily practice [19].

### Strengths and limitations

In this explorative study, an innovative theory- and evidence-based quality-improvement program was developed and implemented in daily physical therapy practice. Integrating multiple proven quality-improvement interventions, combined and informed by outcome data, clearly contributed to the inventive character of this program. Using a participatory strategy for the evaluation of program feasibility during the implementation enhanced the evaluation relevance, as well as providing valuable information regarding the program’s beneficial features and suggestions for improvements from the direct perspectives of the intended end-users. Using both qualitative and quantitative data in a concurrent triangulation design also contributed to the rigor of this study.

This study has several limitations. First, although we intended to include three peer groups for data saturation, only two were ultimately recruited. This could have impacted the validity and transferability of the results. Despite this, the two peer groups provided us with rich data that were deemed sufficient for program evaluation and feasibility study purposes [54]. Second, the peer groups were comprised of physical therapists selected based on the amount of data they collected. As all participants needed to meet external requirements regarding data collection, they could be seen as early adopters. The voluntary participation and external motivation of the participants may have influenced the results and may limit generalizability to other physical therapists. Third, indicative of its exploratory nature, the study’s sample size was limited, but was deemed sufficient to address our research questions. Additionally, the gender distribution among participants, with nine out of 10 being male, does not reflect the typical gender distribution in primary care physiotherapy in the Netherlands. This discrepancy was unintentional, emerging from the recruitment process, but could nevertheless constitute a selection bias, and underscores the need for caution when generalizing findings across diverse physiotherapy contexts. Lastly, although the coach promoted a safe environment during the group meetings, they were not anonymous, and participants may have felt unable to talk openly. Alongside the fact that the assessments could not be blinded, this may have introduced social desirability bias.

### Implications for research and practice

Our findings can be used by national physical therapist bodies and other stakeholders in the field to develop initiatives for improving outcome-based care. This program is well suited for use in primary physical therapy care as it integrates with the peer assessment methodology commonly used in many practices. Such integration minimizes the opportunity costs usually associated with new initiatives by leveraging existing peer-learning and feedback structures, making it a feasible and cost-effective strategy for quality improvement [55]. Additionally, recommendations for advancing the national clinical data registry may further improve the usability for end-users and future researchers, who may wish to study whether the findings are also generalizable to other primary care physical therapist practices. In this study, feedback reporting appeared to support the establishment of quality-improvement goals, and future research could investigate the value of these strategies in evaluating results and changing clinical practices. The sustainability of the observed participant’s behavioral changes and their translation of their revised data-collection routines into quality improvements in care require further consideration. Future studies could improve the program’s feasibility by directly addressing the identified facilitators. Additionally, the program’s impact on patient outcomes should be explored in a full-scale study with long-term follow up.

## Conclusion

This explorative study demonstrated that a quality-improvement program incorporating feedback, peer assessment, and self-assessment in a Plan-Do-Study-Act cycle, and using health outcomes from a national registry, was deemed feasible for quality improvement. The implementation of the program led to knowledge development, perceived improvements in clinical performance, and a change in the behavior of the physical therapists regarding data handling in their routine practice.

### Electronic supplementary material

Below is the link to the electronic supplementary material.

Supplementary Material 1

Supplementary Material 2

Supplementary Material 3

Supplementary Material 4

Supplementary Material 5

Supplementary Material 6

## Data Availability

The datasets used and/or analyzed during the current study are available from the corresponding author upon reasonable request.

## Abbreviations

AIM: Acceptability Intervention Measure
CFN: Cooperation of Physical Therapists Nijmegen
EHR: Electronic health system
FIM: Feasibility Intervention Measure
GPE-DV: Global Perceived Effect
IAM: Intervention Appropriate Measure
KNGF: Royal Dutch Society for Physical Therapy
NSLBP: Nonspecific low back pain
NPRS: Numeric Pain Rating Scale
PROMs: Patient-reported outcome measures
PROs: Patient-reported outcomes
PSFS: Patient Specific Functioning Scale
QBPDS: Quebec Pain Disability Scale
SBST: STarT Back Screening Tool
SD: Standard deviation
SQUIRE: QUality Improvement Reporting Excellence
