# Supplementary material for: Feasibility of a quality-improvement program based on routinely collected health outcomes in Dutch primary care physical therapist practice: a mixed-methods study
Source: BMC Health Serv Res. 2024 Apr 24;24:509. doi: 10.1186/s12913-024-10958-5 (PMC11040789; doi:10.1186/s12913-024-10958-5)
Supplement: Supplementary file 1 — Supplementary Material 1 [file 12913_2024_10958_MOESM1_ESM.docx]

**Supplementary file 2 | Process and outcome indicators of the PROMs for patients with NSLBP**

| **Type of indicator** | **PROM** | **Domain** | **Quality indicator description** |
| --- | --- | --- | --- |
| Process | NPRS | Pain intensity | The percentage of patients with NSLBP who received physical therapy treatment and who completed the NPRS before and after treatment to evaluate pain intensity |
| Process | PSFS | Physical activity | The percentage of patients with NSLBP who received physical therapy treatment and who completed the PSFS before and after treatment to evaluate physical activity |
| Process | SBST | Profile grouping | The percentage of patients with NSLBP who received physical therapy treatment and who completed the SBST before treatment |
| Process | QBPDS | Physical functioning | The percentage of patients with NSLBP who received physical therapy treatment and who completed the QBPDS before and after treatment to evaluate physical function |
| Process | GPE-DV | Perceived effect | The percentage of patients with NSLBP who received physical therapy treatment and who completed the GPE-DV after treatment |
| Change | NPRS | Pain intensity | The mean change score^a^ of patients with NSLBP who received physical therapy treatment and were measured with the NPRS before and after treatment to evaluate pain intensity |
| Change | PSFS | Physical activity | The mean change score^a^ of patients with NSLBP who received physical therapy treatment and were measured with the PSFS before and after treatment to evaluate physical activity |
| Change | QBPDS | Physical functioning | The mean change score^a^ of patients with NSLBP who received physical therapy treatment and were measured with the QBPDS before and after treatment to evaluate physical functioning |
| Score (average) | GPE-DV | Perceived effect | The mean score of patients with NSLBP who received physical therapy treatment and are measured with the GPE-DV after the treatment |

NPRS = Numeric Pain Rating Scale; NSLBP = nonspecific low back pain; PROM = patient-reported outcome measure; PSFS = Patient Specific Functional Scale; SBST = STarT Back Screening Tool; QBPDS = Quebec Back Pain Disability Scale; GPE-DV = Global Perceived Effect—Dutch Version.

^a^Data exported from the national data registry do not allow for the calculation of 95% confidence intervals.

*Note*: Reprinted from Verburg AC, van Dulmen SA, Kiers H, Nijhuis-van der Sanden MW, van der Wees PJ. Patient-Reported Outcome-Based Quality Indicators in Dutch Primary Care Physical Therapy for Patients With Nonspecific Low Back Pain: A Cohort Study 2021(4)
