# Supplementary material for: Feasibility of a quality-improvement program based on routinely collected health outcomes in Dutch primary care physical therapist practice: a mixed-methods study
Source: BMC Health Serv Res. 2024 Apr 24;24:509. doi: 10.1186/s12913-024-10958-5 (PMC11040789; doi:10.1186/s12913-024-10958-5)
Supplement: Supplementary file 2 — Supplementary Material 2 [file 12913_2024_10958_MOESM2_ESM.docx]

**Supplementary File 1 | Quality-improvement program content**

*Step 1. E-learning*

The participants were offered the opportunity to complete the e-learning module ‘How do I use data in my practice’ from the Dutch Institute of Allied Health Care (25), which takes approximately six hours to complete.

*Step 2. Data extraction 1*

All participants received instructional hand-outs on how to examine and obtain outcomes from the national clinical data registry. Next, participants extracted personal data exports (data period January 2021 to December 2021) with process and outcome indicators of PROMs for patients with NSLBP (see Additional File 1)(3,4).

*Step 3. Feedback report on process and outcomes of care 1*

Based on the received data exports, a feedback report (see Additional File 3) was created by the researcher (LS), in which the indicators from all participants forming a peer group were anonymously presented. This included charts, bar charts, radar plots, and numerical tables with benchmark data from the national registry (26–28).

*Step 4. Critical reflection on data by peer assessment 1*

A peer assessment meeting, facilitated by an experienced coach (RvH), marked the actual outset of the quality-improvement cycle (15,20,29). During this meeting, participants discussed the feedback reports from step 3 and drafted a rapid improvement Plan-Do-Study-Act cycle (see Additional File 4), including individual quality-improvement goals (6,30,31), informed by both discussing their processes and the outcome data as presented in the reports. In addition, they were asked about their attitudes regarding data handling during their daily practice and their learning experiences developed by participating in the program thus far.

*Step 5. Self-assessment 1*

To encourage involvement in the quality-improvement cycle, participants performed a self-assessment of their clinical performance halfway through the program, during which they rated their perceived progress towards reaching their quality-improvement goals (32). The progress scores were an integer from 1 (no development) to 10 (maximum development) (32).

*Step 6. Data extraction 2, feedback report 2, and peer assessment 2*

Another peer assessment meeting, led by the same coach (RvH), was organized at the end of the quality-improvement cycle. Prior to the meeting, the participants again extracted their personal data exports (data period January 2022 to March 2022) and a second feedback report was created, which allowed for a comparison of the processes and outcome indicators between the two time periods. During the second meeting, the participants again discussed the feedback reports, their attitudes towards goal attainment (Plan-Do-Study-Act cycle), and their experiences regarding the transfer-of-learning to their workplace (behavioral change). Moreover, they were asked about the learning outcomes they anticipated when moving along the quality-improvement cycle, and they reflected on critical success features and future advancements of the program.

*Step 7. Self-assessments 2 and 3*

Participants again performed self-assessments of their clinical performance three and nine months after the end of the program.

*Step 8. Data extraction 3*

Participants were asked again to extract their personal data exports (data period April 2022 to September 2022) from the national clinical registry nine months after the end of the program.
