# Supplementary material for: Feasibility of a quality-improvement program based on routinely collected health outcomes in Dutch primary care physical therapist practice: a mixed-methods study
Source: BMC Health Serv Res. 2024 Apr 24;24:509. doi: 10.1186/s12913-024-10958-5 (PMC11040789; doi:10.1186/s12913-024-10958-5)
Supplement: Supplementary file 3 — Supplementary Material 3 [file 12913_2024_10958_MOESM3_ESM.docx]

**Supplementary file 6 | Script for peer assessment meeting 2**

Each meeting will comprise one peer group consisting of five physical therapists. The meetings for each peer group will last up to two hours, with peer group 2 meeting immediately after peer group 1. Both meetings will be held on Friday 22 April 2022 between 10:00 and 12:00 (peer group 1) and 12:15 and 14:15 (peer group 2), at the practice of Fysiotherapie Fy-fit, Nijmegen, the Netherlands. Both meetings will be led by a coach experienced in mentoring peer-assessment meetings and group discussions.

Prior to the meeting, all participants will receive:

- Visual feedback report 2 (selected data period of January 2022 to March 2022), allowing for a comparison with the processes and outcome indicators in report 1.
- Plan-Do-Study-Act cycle forms with their personal quality-improvement goals and action plan as formulated at the end of meeting 1.
- Informed consent (audio and video recording).
- Script with accompanying questions to support subgroup discussions during the meeting.

**The meeting will consist of:**

1. *Kick-off plenary (10 min)*

- Welcome words and an outline of the design of the upcoming session.

1. *In separate subgroups (40 min)*

All participants will bring the visual feedback reports and their Plan-Do-Study-Act cycles with personal quality-improvement goals to their subgroup:

- One group of three and one group of two participants will be formed. The subgroups will review the visual feedback reports and Plan-Do-Study-Act cycles. The following questions will be used by participants to guide the discussion:
- How do the data differ from those presented in visual feedback report 1?
- Could these differences be explained by changes in my clinical behavior?

Subsequently, peers will discuss their differences and similarities, and how they can learn from each other, with the following questions as a guideline:

- To what extent have the formulated quality-improvement goals been accomplished?
- Why were the formulated quality-improvement goals (partly) accomplished or not accomplished? What are the underlying factors?
- To what extent is a successful transfer-of-learning (into routine practice) accomplished?
- Which results were obtained and what is the perceived value of these results (related to quality of care)?

1. *Short break (5 min)*
2. *Sequel plenary (30 min)*

- The findings and insights of the subgroups will be further discussed in the plenary, with the goal of initiating a discussion at the peer-group level. What the peers learned from each other will be discussed using the abovementioned topics.

The following aspects will be discussed in further detail:

- Changes in clinical behavior, from intended behavioral changes formulated during meeting 1 to actual behavioral changes.
- Barriers and facilitators to obtaining behavioral changes.
- The level of goal attainment, with special attention paid to data collection routines.

Additionally, the anticipated learning outcomes will be questioned in the plenary:

- What are the most important learning outcomes participants anticipate from participating in the quality-improvement program?
- Which components are perceived as successful features and how should the program be improved in the future?

1. *Closing plenary (5 min)*

- Acknowledgment of participation
