# Supplementary material for: Feasibility of a quality-improvement program based on routinely collected health outcomes in Dutch primary care physical therapist practice: a mixed-methods study
Source: BMC Health Serv Res. 2024 Apr 24;24:509. doi: 10.1186/s12913-024-10958-5 (PMC11040789; doi:10.1186/s12913-024-10958-5)
Supplement: Supplementary file 4 — Supplementary Material 4 [file 12913_2024_10958_MOESM4_ESM.docx]

**Supplementary file 3 | Example of Visual Feedback Report**

**Bar-chart**

**Radar-plot**

**
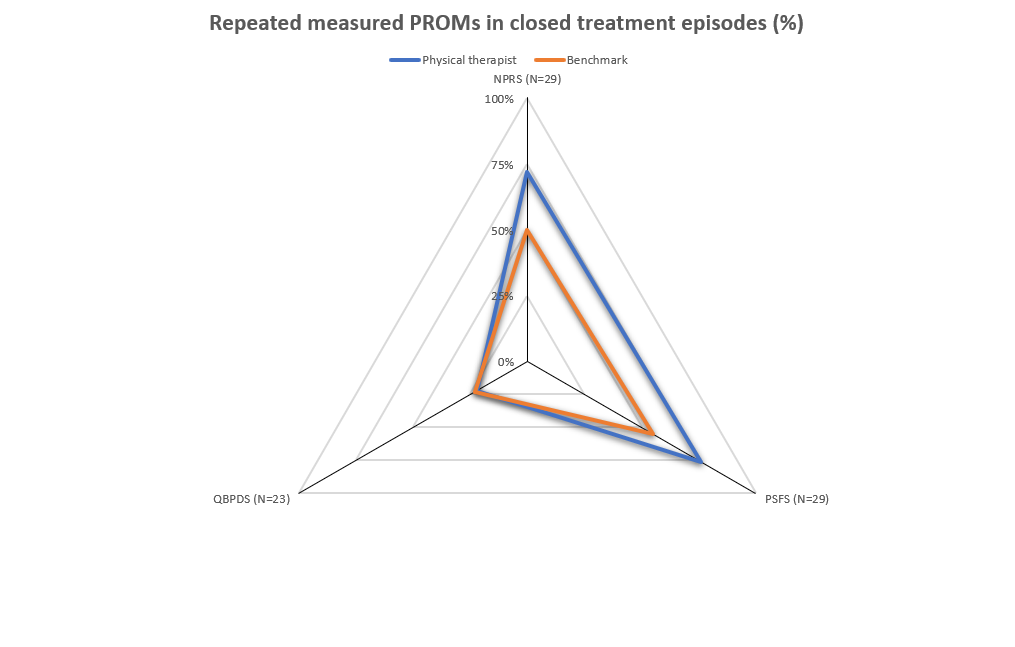
**

**Chart**

**
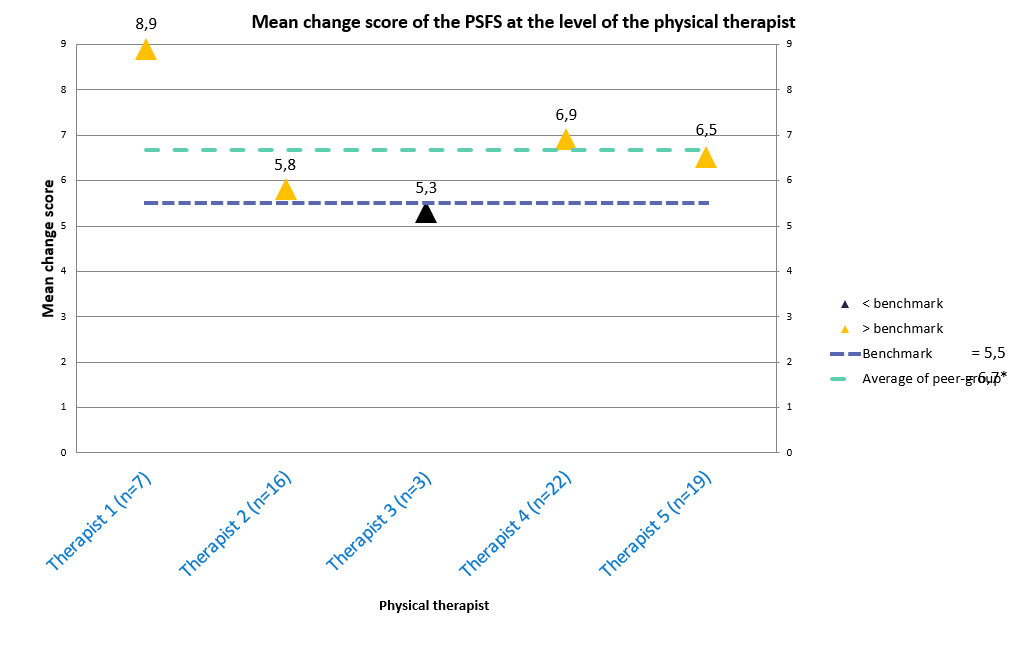
**

**Numerical Table**

**
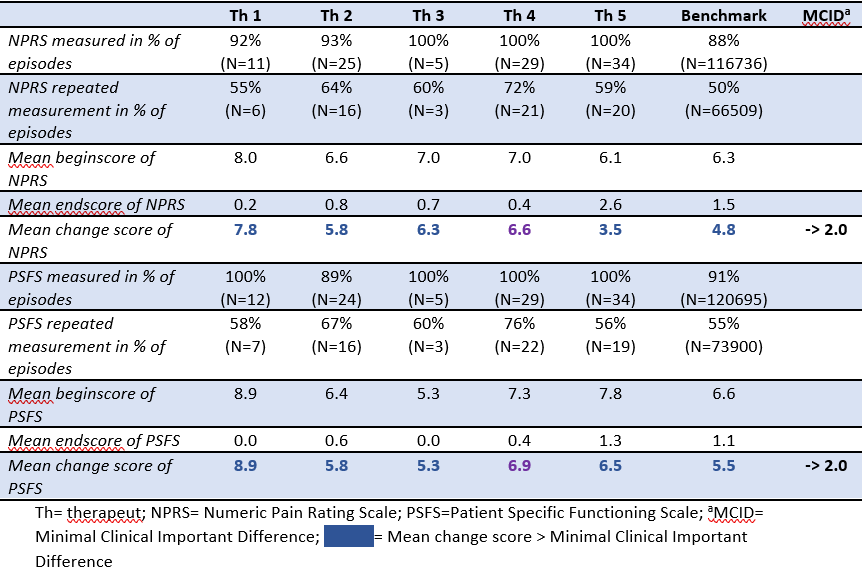
**
