# Supplementary material for: Feasibility of a quality-improvement program based on routinely collected health outcomes in Dutch primary care physical therapist practice: a mixed-methods study
Source: BMC Health Serv Res. 2024 Apr 24;24:509. doi: 10.1186/s12913-024-10958-5 (PMC11040789; doi:10.1186/s12913-024-10958-5)
Supplement: Supplementary file 5 — Supplementary Material 5 [file 12913_2024_10958_MOESM5_ESM.docx]

**Supplementary file 4 | PDSA cycle format**

| **PDSA cycle**  *Quality improvement cycle: collecting, interpretating and using health outcomes*  Name:   \|  \| \| --- \|   Date:   \|  \| \| --- \| |
| --- | --- | --- |


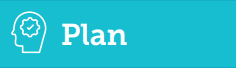


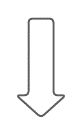


| **PLAN** | |
| --- | --- |
| ***What*** *are your personal quality improvement goals?* | |
|  | Date |
|  |  |
|  |  |
|  |  |


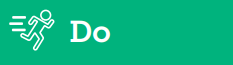


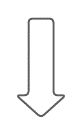


| **DO** | | |
| --- | --- | --- |
| ***What*** *are you going to do to achieve formulated goals?* | | |
| Date Responsible/involved | | |
|  | - | - |
|  | - | - |
|  | - | - |


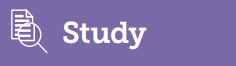


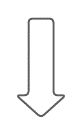


| **CHECK** | | |
| --- | --- | --- |
| ***How*** *are you going to evaluate the development in reaching formulated goals and the effects?* | | |
| Evaluation date Responsible/involved & result | | |
|  | - | - |
|  | - | - |
|  | - | - |


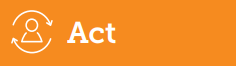


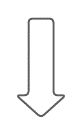


| **DO** | | |
| --- | --- | --- |
| ***Which*** *adjustments are necessary to achieve formulated goals?* | | |
| Date Responsible/involved | | |
|  |  |  |
|  |  |  |
|  |  |  |
