# Supplementary material for: Feasibility of a quality-improvement program based on routinely collected health outcomes in Dutch primary care physical therapist practice: a mixed-methods study
Source: BMC Health Serv Res. 2024 Apr 24;24:509. doi: 10.1186/s12913-024-10958-5 (PMC11040789; doi:10.1186/s12913-024-10958-5)
Supplement: Supplementary file 6 — Supplementary Material 6 [file 12913_2024_10958_MOESM6_ESM.docx]

**Supplementary file 5 | Script for peer assessment meeting 1**

Each meeting will comprise one peer group consisting of five physical therapists. The meetings for each peer group will last up to two hours, with peer group 2 meeting immediately after peer group 1. Both meetings will be held on Friday 21 January 2022 between 10:00 and 12:00 (peer group 1) and 12:15 and 14:15 (peer group 2), at the practice of Fysiotherapie Fy-fit, Nijmegen, the Netherlands. Both meetings will be led by a coach experienced in mentoring peer-assessment meetings and group discussions.

Prior to the meeting, all participants will receive:

- A visual feedback report (selected data period of January 2021 to November 2021).
- Two Plan-Do-Study-Act cycle forms. On one form, participants can design their preliminary and emerging quality-improvement goals and action plan during the meeting. On the other form, participants will note their final quality-improvement goals and action plan no later than one week after the meeting and send it to the research team.
- Informed consent (audio and video recording).

**The meeting will consist of:**

1. *Kick-off plenary (20 min)*

- Welcome words
- Determine whether participants completed the e-learning and delve into their (learning) experiences and thoughts regarding the e-learning.
- Examine how participants perceived reviewing their own individual data in the national clinical registry and creating a personal export file.
- Evaluate how participants perceived receiving a visual feedback report and their opinion regarding the content and comprehensibility.

1. *In separate subgroups (30 min)*

- One group of three and one group of two participants will be formed. The subgroups will conduct more substantive and in-depth discussions of the visual feedback reports. The following questions will be used by participants to guide the discussion:
- What do I see and what stands out?
- Do I recognize what I see in my own personal data?
- Where are differences in the data of different peer group members?
- Can I/we explain these differences?

1. *Short break (5 min)*
2. *Sequel plenary (30 min)*

- The findings and insights of the subgroups will be further discussed in the plenary, with the goal of initiating a discussion at the peer-group level. The input will again be formed by the abovementioned questions.

1. *In separate subgroups (20 min): developing quality-improvement goals*

- Two subgroups (three and two participants) will be formed. Together with the coach, participants will be encouraged to formulate personal quality-improvement goals using the following questions:
- Based on the discussions, what do I wish to improve?
- How do I translate this to formulating my own personal quality-improvement goals?

1. *Closing plenary (10 min)*

- Findings and formulated quality-improvement goals will be further discussed plenary.
- A WhatsApp group will be formed for each peer group, together with the coach.
